# PSYCHOLOGICAL WELL-BEING AND THE ONSET OF COGNITIVE IMPAIRMENT AMONG CHINESE OLDER ADULTS

**DOI:** 10.1093/geroni/igz038.423

**Published:** 2019-11-08

**Authors:** Jiaan Zhang

**Affiliations:** Fudan University, Shanghai, China

## Abstract

Previous research has shown the beneficial effects of positive psychological assets on health, but more research is needed to confirm the prospective effects on cognitive function. The purpose of this study is to examine the relationship between psychological well-being and the earliest onset of cognitive impairment among Chinese older adults. Data came from 2000 to 2014 waves of the Chinese Longitudinal Healthy Longevity Survey. Study sample consisted of 6,225 older adults who were free from cognitive impairment in 2000. Psychological well-being was measured based on seven items that assessed optimism, conscientiousness, self-determination, happiness, self-esteem, pessimism, and loneliness, with responses ranging from “always (1)” to never (5)”. Negative feelings items were reverse coded. Higher score indicated more positive psychological well-being. Cognitive impairment was measured by a Chinese version of the Mini-Mental State Examination. Respondents scored at or above 24 were regarded as having no cognitive impairment. A multi-category time-varying variable was used to capture four potential outcomes: (1) persistently free of cognitive impairment between waves, (2) onset of cognitive impairment, (3) death between waves, and (4) attrition. Socio-demographics, chronical diseases conditions, functional health status were served as controls. Multilevel multinomial logistic regression models that account for clustering of observations within a subject over time were employed for the study. Results show that more positive psychological well-being is significantly associated with reduced risk of cognitive impairment onset and death over time. Results suggest that developing more psychological resilience-based intervention programs among older adults may help them delay the onset of cognitive impairment.

